# HER2-targeted therapies in cancer: a systematic review

**DOI:** 10.1186/s40364-024-00565-1

**Published:** 2024-02-02

**Authors:** Kunrui Zhu, Xinyi Yang, Hebei Tai, Xiaorong Zhong, Ting Luo, Hong Zheng

**Affiliations:** 1 Institute for Breast Health Medicine, Cance Center, Breast Center, West China Hospital, Sichuan University, Chengdu, 610041 Sichuan China; 2 College of Clinical Medical, Guizhou Medical University, Guiyang, 550000 Guizhou Province China

**Keywords:** HER2, Targeted therapy, Antibody-drug conjugates

## Abstract

Abnormal alterations in human epidermal growth factor receptor 2 (HER2, neu, and erbB2) are associated with the development of many tumors. It is currently a crucial treatment for multiple cancers. Advanced in molecular biology and further exploration of the HER2-mediated pathway have promoted the development of medicine design and combination drug regimens. An increasing number of HER2-targeted drugs including specific monoclonal antibodies, tyrosine kinase inhibitors (TKIs), and antibody-drug conjugates (ADCs) have been approved by the U.S. Food and Drug Administration. The emergence of ADCs, has significantly transformed the treatment landscape for various tumors, such as breast, gastric, and bladder cancer. Classic monoclonal antibodies and novel TKIs have not only demonstrated remarkable efficacy, but also expanded their indications, with ADCs in particular exhibiting profound clinical applications. Moreover the concept of low HER2 expression signifies a breakthrough in HER2-targeted therapy, indicating that an increasing number of tumors and patients will benefit from this approach. This article, provides a comprehensive review of the underlying mechanism of action, representative drugs, corresponding clinical trials, recent advancements, and future research directions pertaining to HER2-targeted therapy.

## Introduction

Human growth factor receptor 2(HER2/erbB2) belongs to the epidermal growth factor receptor(EGFR) family and comprises four members: erbB1(EGFR/HER1), erbB2(HER2), erbB3(HER3), and erbB4(HER4) [1]. These family members mainly consist of three domains: an extracellular domain (ECD), a transmembrane domain (TMD), and an intracellular region [1]. The ECD consists of four subdomains (I-IV) [2]. The ECD adopts a closed conformation involving domains II and IV in the absence of ligand. Ligand binding between domains I and III untethers the dimerization arm in domain II, leading to receptor homodimers or heterodimerization, allosteric kinase activation, and downstream signaling [3]. Overexpressing of HER2 proteins facilitates the formation of either homodimers or heterodimers [4]. Therefore promoting growth and proliferation are promoted primarily through the MAPK/ERK and PI3K/AKT/mTOR pathways [5].

### HER2 alterations in diverse cancers

Alterations in HER2 expression in tumor cells primarily arise from the mutation, amplification or overexpression of the HER2 gene (erbB2). Mutations in HER2 are most commonly observed within the intracellular tyrosine kinase structural domain, encompassing exon 20 (20%), exon 19 (11%) and exon 21 (9%). The mutation hotspot varies across different types of cancer [6]. The presence of HER2 mutations does not always coincide with HER2 amplification [7], which is often associated with the overexpression of the HER2 protein [8]. HER2 amplification and overexpression have been observed in various types of cancers, including but not limited to breast cancer, gastric cancer, non-small cell lung cancer (NSCLC), bile duct cancer, bladder cancer, and colorectal cancer [9–13]. This association may indicate an unfavorable prognosis or the development of drug resistance in the treatment of various malignancies [14].

The overexpression of HER2 is associated with aggressive behavior and a poorer prognosis in patients with breast cancer and bladder cancer [9, 15–17]. Additionally, amplification or overexpression of the HER2 gene is linked to unfavorable clinicopathological features and prognosis in biliary tract cancers [18]. HER2 alterations have been identified as oncogenic drivers in lung cancer, all of which are correlated with an unfavorable prognosis [19]. While the prognostic significance of HER2 in gastric/gastroesophageal junction adenocarcinoma and colorectal cancer remains debated, some studies suggest that HER2 amplification may be indicative of a poor prognosis [20–23]. However, the QUASAR,FUCOS,and PICCOLO trials have shown no significant correlation between overall survival(OS) or progression-free survival(PFS) in patients with HER2-amplified mCRC [24]. However, studies by Sarah B Fisher [25] and Shen et al. [26] indicated no significant relationship between HER2 expression and gastric cancer prognosis. It is important to note that differences in population characteristics included in the studies may account for the discrepancy in findings [27]. Furthermore, the frequency of amplification is greater among patients with KRAS/BRAF wild-type mutations than among other patients [28, 29]. Patients with either HER2-amplified mCRC or those who have exon 20 insertions(ex20ins) may also face a greater risk of developing brain metastases [29].

### HER2-targeted therapies

**Fig. 1:**
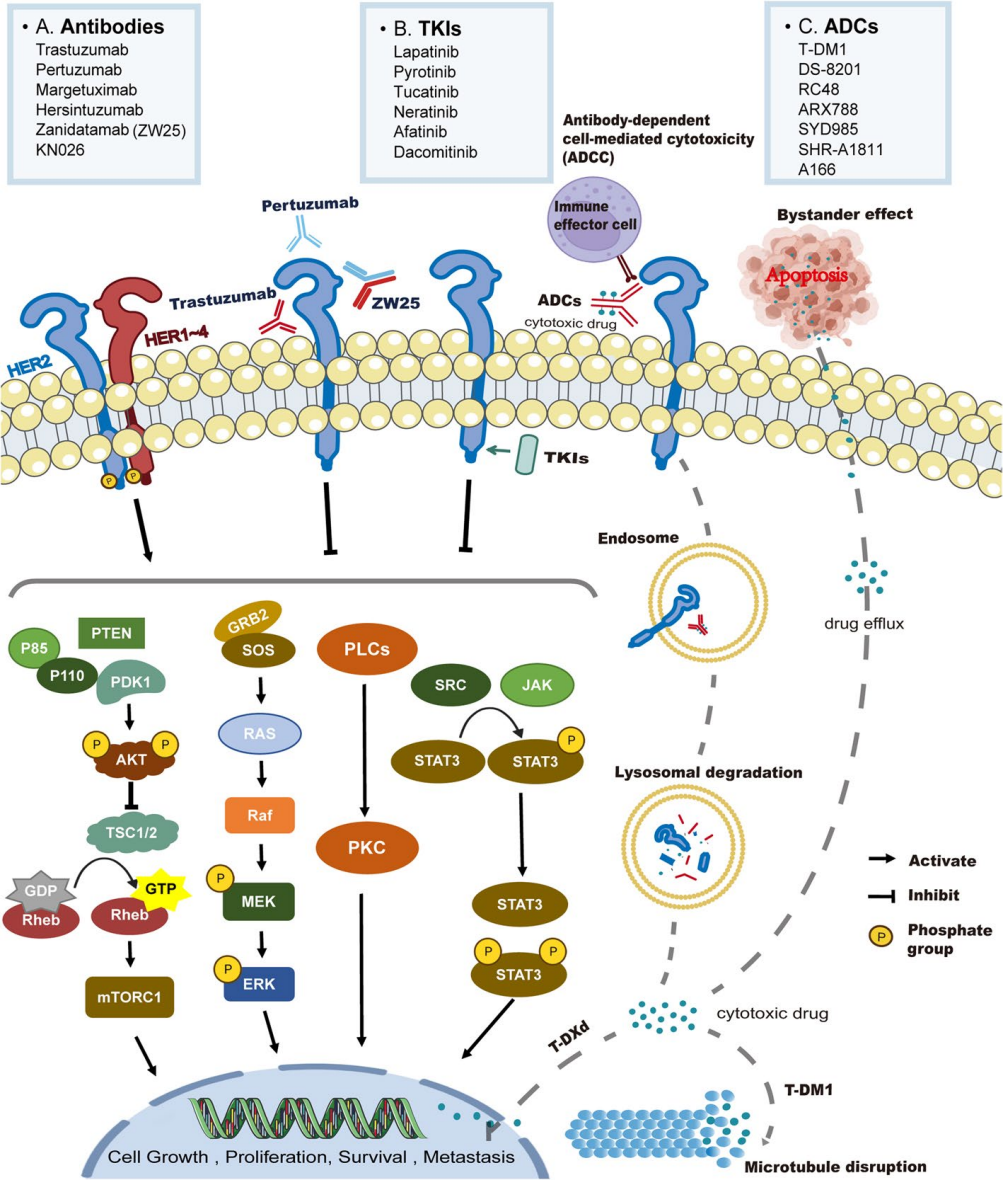
Classification and mechanisms of prevalent anti-HER2-targeting medications. (PTEN: phosphatase and tensin homolog; PDK1: phosphoinositide-dependent protein kinase 1; AKT: protein kinase B; TSC1/2: tuberous sclerosis complex 1/2; GTP: guanosine triphosphate; GDP: guanosine diphosphate; mTORC: mammalian target of rapamycin; GRB2: growth factor receptor-bound protein 2; SOS: guanosine release protein; MEK: mitogen-activated extracellular signal-regulated kinase; ERK:extracellular signal-regulated kinase; PLC: phospholipase C; PKC: protein kinase C; STAT: signal transducer and activator of transcription)

The current classification of HER2-targeted drugs includes antibodies, tyrosine kinase inhibitors(TKIs) and antibody-drug conjugates (ADCs) (Fig. 1). The mechanism of action of antibodies involves two primary aspects: binding to the extracellular domain of the HER2 protein, preventing the formation of HER2-containing heterodimers, modulating the downstream effectors of ERBB2 signaling, and recruiting extracellular immune cells for antibody-dependent cell-mediated cytotoxic effects (ADCCs) [30–32]. Representative drugs include trastuzumab, pertuzumab and ZW25. ZW25, a bispecific antibody, can target both HER2 extracellular region II (pertuzumab binding site) and IV (trastuzumab binding site) and activate ADCC [33–36].

Unlike antibodies, small molecule tyrosine kinase inhibitors (TKIs) can Unlike catalyze the kinase structure within cells and compete with ATP to inhibit the downstream signaling of the HER2 family [37]. TKI drugs block the phosphorylation of tyrosine kinase residues in the PI3K/AKT and MAPK pathways, which regulate tumor cell proliferation, migration, angiogenesis, drug resistance, and apoptosis [38]. In addition, TKI drugs with small molecular weights and high lipid solubilities can effectively penetrate the blood-brain barrier [39], which makes them promising for application in tumors with brain metastases.

ADCs are composed of an antibody, a linker and a cytotoxic small molecule, as exemplified by T-DM1, RC48, and DS-8201 [39]. After the antibody binds to the HER2 protein, the molecules are endocytosed into the cell and subsequently cleaved. The release of cytotoxic small molecules causes damage to DNA, tubulin, or other substances, inhibiting the growth, proliferation, survival, and development of cancer cells [27]. Moreover, these cytotoxic small molecules can diffuse to neighboring target cells and cause nonspecific tumor cell death, known as the bystander effect [40] (Fig. 1). ADCs not only preserve monoclonal antibody efficacy but also enhance cytotoxicity through the incorporation of a small molecule payload, thereby significantly augmenting the therapeutic potential of antitumor drugs [41].

### HER2 targeted therapies in different cancer

#### Breast cancer

##### Antibodies

The introduction of HER2-targeted therapy has revolutionized the landscape of breast cancer treatment. The currently approved HER2 monoclonal antibodies (mAb) for breast cancer treatment include trastuzumab and pertuzumab. Trastuzumab (Herceptin) was the first anti-HER2 humanized mAb developed and was approved for marketing by the U.S. Food and Drug Administration (FDA) in 1998 [33]. Preclinical data demonstrating the synergistic effects of cytotoxic agents and trastuzumab have been obtained, subsequently, many clinical studies have confirmed the powerful therapeutic efficacy of these agents [42–49]. It is currently used for neoadjuvant, adjuvant and advanced salvage therapy for HER2-overexpressed breast cancer. Pertuzumab (Perjeta) was approved for marketing by the FDA in 2012. This is attributed to the findings of several pivotal studies, including the NeoSphere, APHINITY, and CLEOPATRA trials [50–52]. These investigations have demonstrated that the pertuzumab combined with trastuzumab (HP) regimen can further enhance outcomes and prolong survival in patients with HER2-positive breast cancer across neoadjuvant and adjuvant therapy as well as advanced first-line treatment. HP has become an option for neoadjuvant and adjuvant postoperative adjuvant therapy in patients with HER2-positive early breast cancer ≥T2 or ≥N1 and is a standard recommendation for first-line treatment of patients with metastatic breast cancer [53].

##### TKIs

Currently, lapatinib, pyrotinib, tucatinib and neratinib are employed in clinical settings. Lapatinib was approved by the FDA on March 13, 2007 for second-line treatment in patients with advanced or metastatic breast cancer with HER2 overexpression [54]. The updated findings from the ALTERNATIVE Phase III trial demonstrated that in postmenopausal women with HR-positive/HER2-positive advanced breast cancer, the combination of aromatase inhibitors (AIs) with lapatinib significantly extended the median progression-free survival (mPFS) compared to that of patients receiving AI monotherapy (11 vs. 5.6 months, *P* = 0.0063) [54]. The NMPA approved pyrotinib on August 16, 2018, for the treatment of patients with recurrent or metastatic HER2-positive breast cancer who had have not received prior trastuzumab therapy. The PHEDRA and PHILA randomized phase III studies demonstrated the benefits of pyrotinib as a neoadjuvant and advanced first-line therapy for HER2-positive breast cancer [55, 56]. Consequently, in 2020, the Chinese Society of Clinical Oncology (CSCO) has classified pyrotinib as recommended for these patients [57]. In 2020, the FDA approved tucatinib in combination with trastuzumab and capecitabine as a treatment option for patients with advanced HER2-positive breast cancer [58]. These findings are based on the results from the phase III clinical trial (HER2CLIMB) [59]. Neratinib, a dual EGFR/HER2 inhibitor, was FDA-approved for sale on July 17, 2017. Based on the ExteNET study results: patients with HER2 overexpression and high-risk factors after surgery can receive neratinib as intensive adjuvant therapy for one year [60]. This finding highlights the need for intensive anti-HER2 adjuvant therapy in early-stage breast cancer patients at high risk of recurrence.

##### ADCs

Currently, there are two FDA-approved ADCs for the treatment of breast cancer: Trastuzumab Emtansine (T-DM1) and trastuzumab deruxtecan (T-DXd). T-DM1 consists of trastuzumab and the cytotoxic agent mertansine [61]. DS-8201 comprises trastuzumab and a topoisomerase I inhibitor (Deruxtecan) [62]. Compared to T-DM1, T-Dxd has lysable linkers and has a greater drug-to-antibody ratio (8:1), resulting in greater antitumor effects [63]. According to the EMILIA trial [64], TDM1 was associated with an increase in progression-free survival (PFS) to 9.6 months in patients with HER2-positive advanced breast cancer who previously failed trastuzumab and paclitaxel treatment. Furthermore, the DESTINY BREAST03 study confirmed that T-DXd further extended the median PFS to 28.8 months under the same inclusion criteria [63]. The effectiveness of T-DXd in breast cancer patients with low expression of HER2 expression (IHC1+ or IHC2+ and FISH-) was confirmed by the randomized controlled phase III study, DESTINY-Breast04 [65]. This finding provides a novel justification for the precise treatment of patients exhibiting low HER2 expression, thereby compelling the use of FDA-approved T-DXd for the treatment of patients with low HER2 expression in breast cancer.

As of 2021, there are curruntly 34 ADC drugs in clinical trials targeting HER2. Notable examples include SYD985 and RC48. SYD985 is a combination of trastuzumab and docaramazine analogues linked by cuttable connectors(DARs). Preclinical studies have demonstrated that SYD985 has superior antitumor activity compared to T-DM1 [66]. Preliminary results from the CRISTINA SAURA study demonstrated that treatment with SYD985 was effective (ORR of 33% and mPFS of 9.4 months) in HER2-positive metastatic breast cancer patients [67]. Based on these data, the FDA has granted Fast Track designation to SYD985. A Phase III clinical trial, TULIP, is currently underway. RC48-ADC is a domestic original Chinese ADCs consisting of hertuzumab, a histone cleavable linker and monomethyl auristatin E (MMAE) coupling [68]. A phase III clinical trial of RC48 in the treatment of locally advanced or metastatic breast cancer with low expression of HER2 is underway (Tables 1 and 2). ZW-49 is a double-antibody ADC that can target two different sites of ECD4 and ECD2 [69], and is currently undergoing phase I clinical trials. Many promising ADC drug-related studies are underway (Table 2).

**Table 1:** Expression of HER2 in different cancers

HER2 Status Cancer Type	HER2 mutation			Amplification/Frequence(%)	Overexpression(%)	Reference
	Protein Change/Frequence(%)					
Breast cancer	L755S	S310F	V777L	20	15-20	[5, 70–72]
	0.16-0.96	0.16-0.94	0.16-0.92			
Gastric cancer	S310F	S310Y	V842I	11–16	20	[73–78]
	0.19-0.92	0.21-0.89	0.35-0.84			
NSCLC	S310F	D277Y	G776delinsVC	2–3	2.5	[79, 80]
	0.18-0.74	0.54-0.72	0.49-0.71			
Bladder cancer	R678Q	L313V	T733I	8.6	12.4-30	[74, 81, 82]
	0.41-0.99	0.97	0.89-0.94			
Biliary tract cancer	S310F	S310Y	D769Y	3–19	5-15	[18, 83–88]
	0.27-0.96	0.24-0.94	0.23-0.83			
Colorectal cancer	S310F	M1014K	T862A	2-5.8	5	[89–91]
	0.24-0.89	0.77	0.73

**Table 2:** Recent agents targeting HER2 in different cancer

Cancer Type Drug			Breast cancer	Gastric cancer	NSCLC	Bladder cancer	Biliary tract cancer	Colorectal cancer
		Indication	HER2 overexpressing (Adjuvant, neoadjuvant and Late first-line therapy) [92] (FDA/EMA Approved)	HER2-overexpressing metastatic gastric/Gastroesophageal junction adenocarcinoma (Adjuvant, first - and second-line therapy) [93] (FDA/EMA Approved)	_	_	_	_
		Ongoing clinical trials (stage)	NCT02625441 (III) NCT02625441(III) NCT03084939(III)	NCT03615326 (III) NCT04661150 (II) NCT04888663 (II)	NCT04644237 (II) NCT03845270(II) NCT04579380(II)	NCT02091141 (II) NCT05786716 (II/III)	NCT02091141(II) NCT04579380(II) NCT05749900(I/II)	NCT03457896 (II) NCT03043313 (II) NCT05786716(II/III)
	Pertuzumab	Indication	HER2-overexpressing (Adjuvant, neoadjuvant and Late first-line therapy) [94] (FDA/EMA Approved)	_	_	_	_	_
		Ongoing clinical trials (stage)	NCT02625441 (III) NCT03493854(III) NCT01358877(III)	NCT01461057 (III) NCT02581462 (II/III)	NCT02507375(I) NCT00855894 (II) NCT00063154 (II)	NCT02091141 (II) NCT05786716 (III) NCT02465060(II)	NCT02091141(II) NCT05786716(II/III)	NCT03365882(II) NCT02465060(II) NCT01376505(I)
	Inetetamab	Indication	HER2-overexpressing (Late first-line therapy, second and third line therapy) [95] (NMPA Approved)	_	_	_	_	_
		Ongoing clinical trials (stage)	NCT04941885(II) NCT04681911(II) NCT05764941(real-word study)	_	NCT05016544(I)	_	_	_
	Margetuximab	Indication	HER2-overexpressing breast cancer(Third-line therapy) [96](FDA Approved)	_	_	_	_	_
		Ongoing clinical trials (stage)	NCT04425018(II) NCT04262804(II) NCT04425018(II)	NCT04082364(II) NCT01148849(I)	NCT03219268(I)	_	_	_
ADC	T-DM1	Indication	HER2-overexpressing breast cancer(Adjuvant, neoadjuvant and Second-line therapy) [97](FDA/EMA Approved)	_	_	_	_	_
		Ongoing clinical trials (stage)	NCT03529110 (III) NCT03084939(III) NCT04740918(III)	NCT02465060(II)	NCT04042701(I) NCT05650879 (I) NCT02314481(II)	NCT02465060(II) NCT02675829(II)	NCT02465060(II)	NCT02465060(II) NCT05578287 (II) NCT03225937 (II)
	T-DXd	Indication	HER2-overexpressin(Adjuvant, neoadjuvant and Late second-line therapy) [98] HER2-lowexpression (Adjuvant and second-line therapy) [99] (FDA/EMA Approved)	HER2-overexpressing gastric cancer/ Gastroesophageal junction carcinoma (Second-line therapy) [100](FDA/EMA Approved)	HER2-positive metastatic (Adjuvant) [101] (FDA Approved)	_	_	_
		Ongoing clinical trials (stage)	NCT04622319 (III) NCT04784715 (III) NCT04494425 (III)	NCT04639219 (II) NCT04989816 (II) NCT04379596 (II)	NCT04686305(I) NCT05246514(II) NCT05048797 (III)	NCT04482309(II) NCT04644068(I/II	NCT04482309(II) NCT04644068(I/II)	NCT04744831(II) NCT04639219(II) NCT04644068(I/II)
	RC-48	Indication	_	HER2-overexpressing metastatic gastric/ Gastroesophageal junction adenocarcinoma(Second and third line therapy therapy) (NMPA Approved)	_	HER2-overexpression urothelial carcinoma(Second-line therapy) (NMPA Approved)		_
		Ongoing clinical trials (stage)	NCT03052634(I/ II) NCT05134519 (II) NCT05331326 (II)	NCT05514158(I) NCT04714190 (III)	NCT04311034(Ib) NCT05745740(I)	NCT05356351(II) NCT05297552(II) NCT05016973(II)	NCT04329429(II) NCT05417230(II)	NCT05785325(II) NCT05578287(II)
TKI	Neratinib	Indication	HER2 -overexpressing (Adjuvant ,Second - and third-line therapy) [102] (FDA/EMA Approved)	_	_	_	_	_
		Ongoing clinical trials (stage)	NCT05760612 (III) NCT04965064(II) NCT05252988(II)	NCT05512182(II) NCT05274048(I)	NCT01827267(II)	_	NCT03919292(I)	NCT01960023(II) NCT03457896(II) NCT03919292(I)
	Lapatinib	Indication	HER2 -overexpressing breast cancer (Second - and third-line therapy) [103] (FDA/EMA Approved)	_	_	_	_	_
		Ongoing clinical trials (stage)	NCT05122494 (III) NCT03084939(III) NCT00770809(III)	NCT00680901 (III) NCT02015169(II) NCT00313599(I)	NCT01306045(II) NCT03845270(II) NCT01184482(I)	NCT00313599(I) NCT00623064(I)	NCT01184482(I)	NCT04831528(II) NCT00044343(II) NCT01184482(I)
	Tucatinib	Indication	HER2 -overexpressing (Adjuvant, neoadjuvant, Second - and third-line therapy) [104] (FDA/EMA Approved)	_	_	_	_	_
		Ongoing clinical trials (stage)	NCT03054363 (I/ II) NCT02614794(II) NCT05132582(III)	NCT05190445(II) NCT05382364(I) NCT02892123(I)	NCT04579380(II) NCT02892123(I)	_	NCT04579380(II)	NCT05253651(III) NCT03043313 (II) NCT04430738 (II)
	Pyrotinib	Indication	HER2 -overexpressing (Adjuvant, neoadjuvant and Late first-line therapy) [105] (NMPA Approved)	_	_	_	_	_
		Ongoing clinical trials (stage)	NCT04646759(III) NCT04254263(III) NCT02973737(III)	NCT05070598(II) NCT05111444(II) NCT02500199(I)	NCT04144569(II) NCT04447118(III) NCT05751018(III)	NCT05318339(II)	NCT04571710(II)	NCT05350917(II)

#### Gastric cancer

##### Antibodies

Gastric cancer is a prevalent malignancy that ranks fifth in terms of global incidence. Regrettably, the prognosis for advanced or metastatic gastric cancer remains dismal, with a mere 5% to 10% five-year survival rate [106]. Based on the results of the phase III clinical study ToGA, the FDA approved trastuzumab for treating HER2-overexpressing gastric/gastroesophageal junction(G/GEJ) adenocarcinoma in January 2010 [107]. In the phase III clinical trial KEYNOTE-811, pembrolizumab plus trastuzumab resulted in a significant reduction of tumor size and improved objective remission rates in patients with HER2-positive adenocarcinoma of the G/GEJ adenocarcinoma (ORR 74.4%) [108]. A phase II trial demonstrated that using trastuzumab/trastuzumab+pertuzumab combination chemotherapy during the perioperative phase increased the pathologic response rate from 25% to 45% [109]. An ongoing extension phase III clinical trial (INNOVATION), is currently underway. Margetuximab, a monoclonal antibody optimized for the Fc structural domain, has demonstrated improved ADCC effects and antitumor immune activity. In the phase II trial, margetuximab was combined with pablizumab as a second-line treatment for HER2-positive (and/or PD-L1 positive) patients with gastroesophageal cancer (GEA), which resulted in significant improvement in patient survival (HER2-positive: ORR 28.2%, DCR 63.4%, mPFS 4.3 months, mOS 13.9 months) [110]. As a result of these findings, the FDA has approved the use of margetuximab for the treatment of adenocarcinoma of GEA. A phase IB/II trial revealed that combining trastuzumab with the ramucirumab (VEGF-2-targeting agent) improved the prognosis of patients with gastric cancer who had progressed after trastuzumab treatment (mPFS 7.2 months, ORR 33%, DCR 95.6%) [111].

Zanidatamab (ZW25) and KN026 are considered promising bispecific antibodies that target HER2 for the treatment of gastric cancer. In 2019, Zanidatatamab was granted fast track status by the FDA due to its demonstrated efficacy when combined with standard chemotherapy in the first-line treatment of advanced GEA (ORR 54%, DCR 79%) [112]. In a previous phase I clinical trial, KN026 was administered to patients with adenocarcinoma and HER2 overexpression in the gastroesophageal junction, revealing an objective response rate of 55.6% and manageable adverse reactions [113]. Several ongoing clinical trials investigating KN026 and ZW25 for advanced/metastatic gastric cancer hold great promise for their results.

##### TKIs

Although TKIs have demonstrated efficacy in the treatment of breast cancer, their effectiveness in treating gastric cancer is remians limited. The combination of lapatinib with chemotherapeutic agents, as observed in the tytan and logic trials, did not result in improved overall survival in patients with HER2-positive gastroesophageal adenocarcinoma [114, 115]. This lack of improvement may be attributed to factors such as lapatinib's toxicity, and patient demographic factors, including age and region. While afatinib and tucatinib have shown some antitumor activity in patients with HER2-positive gastroesophageal cancer [116, 117], further research is necessary to determine their specific efficacy.

##### ADCs

The FDA has approved trastuzumab deruxtecan (DS-8201) for use in patients with locally advanced or metastatic HER2-positive G/GEJ adenocarcinoma who have been treated with trastuzumab based on the results of the DESTINY-Gastric01 trial [118]. The results of the second-phase clinical trial DESTINY-Gastric02 demonstrated that T-DXd monotherapy exhibited remarkable efficacy as a second-line treatment for patients with HER2-positive advanced gastric cancer(ORR 41.8%, overall survival (OS)>1 year). This is currently the highest recorded outcome among second-line treatment regimens [119]. T-DXd has shown clinical activity in patients with previously treated HER2-low expression (IHC 2+/ISH-, IHC 1+) G/GEJ adenocarcinoma, without any reported new adverse effects [118]. A phase II trial demonstrated positive results in the treatment of locally advanced or metastatic gastric cancer with HER2 IHC 2+/3+ in patients who had undergone two or more prior systemic chemotherapies (ORR 25%, DCR 42%, mOS 7.6 months) [120]. In a phase I trial (ACE-Gastric-01), the novel ADC drug ARX788 exhibited good tolerability and antitumor activity in treating patients with HER2-positive advanced gastric cancer and GEJ adenocarcinoma [91]. ARX788 has several advantages due to its Zanidatamab-specific double antibody components and ADC drugs. Currently, clinical trials are underway to explore the use of ARX788 and RC48.

#### NSCLC

##### Antibodies

HER2-targeting monoclonal antibodies, like trastuzumab and pertuzumab, have not demonstrated significant antitumor activity when used alone in patients with HER2-mutated NSCLC [121–123]. A recent phase I-II study revealed that the combination of trastuzumab and pertuzumab exhibited only modest antitumor activity in advanced HER2-mutated NSCLC patients after multiple treatments [124]. Further investigation are warranted to determine the reasons for the poor efficacy of monoclonal antibody in treating NSCLC.

##### TKIs

Non-selective tyrosine kinase inhibitors (TKIs), such as afatinib, dacomitinib, and neratinib, exhibit poor antitumor activity against non-small cell lung cancer (NSCLC), and their efficacy may be linked to specific HER2 mutation types [125–128]. Selective TKIs such as poziotinib and pyrotinib have demonstrated promising antitumor activity in recent studies. In particular, poziotinib has shown superior activity against NSCLC patients with HER2 exon 20 mutations in ex vivo experiments and several phase II clinical trials (ORR 39%, DCR 73%) [129]. It is important to note that major adverse effects such as rash and diarrhea have been reported [130, 131]. Despite these adverse effects, poziotinib is considered to be one of the most efficacious selective TKIs currently available. Preclinical studies have demonstrated that the combination of poziotinib and T-DM1 results in complete tumor regression [6]. Furthermore, in patients with metastatic NSCLC with HER2 mutation or HER2 amplification, the combination of pyrotinib and apatinib has exhibited favorable efficacy (mPFS 5.8-8.5 months) [132]. In addition to the aforementioned TKIs, tarloxotinib and mobocertinib have shown potential effectiveness in treating NSCLC patients with HER2 mutations [133–135]. However, further research is needed of the preliminary stage of development of these drugs.

##### ADCs

T-DXd has been approved by the FDA for the treatment of patients with advanced NSCLC. Other ADC drugs, such as T-DM1, RC48, and SHR-A1811 are also being explored for drug efficacy and safety. The multicenter clinical phase II trial DESTINY-Lung01 [136] demonstrated that T-DXd had effective antitumor activity in patients with HER2 mutations and overexpression (mPFS 6.4-14 vs. 2.8-7 months) [137]. Subsequently, the follow-up study DESTINY-Lung02 revealed that administering T-DXd at a lower dosage significantly decreased the occurrence of interstitial pneumonia (ILD) and other adverse events, while sustaining an objective response rate (ORR) (5.9% vs. 14% for ILD incidence; 53.8% vs. 42.9% for ORR) [138]. The FDA-approved T-DXd for the backline treatment of patients with advanced NSCLC with HER2 mutations was based on the DESTINY-Lung02 study. The ongoing phase Ib clinical trial DESTINY-Lung03 is investigating the safety and efficacy of T-DXd in combination with immunotherapy for patients with HER2-positive advanced NSCLC. However, T-DM1 has limited efficacy in NSCLC patients, with only a few studies showing mild antitumor activity [131, 139]. Additionally, other types of ADC drugs, such as RC48 and SHR-A1811, are undergoing clinical trials for patients with HER2-abnormal NSCLC.

#### Bladder cancer

##### Antibodies

HER2 monoclonal antibodies are not yet approved for urothelial carcinoma treatment by the FDA. A multicenter phase II trial conducted by Hussain et al. showed that trastuzumab combined with chemotherapy significantly improved outcomes in advanced urothelial carcinoma patients with HER2-positive disease (DFS 9.3 month, OS 14.1 month) [140]. Another phase I/II trial confirmed that trastuzumab combined with radiotherapy + paclitaxel enhanced the treatment efficacy in patients with muscle-invasive urothelial carcinoma who were unsuitable for bladder resection, but these treatment resulted in significant gastrointestinal toxicity (35%) [141].

##### TKIs

Current research on TKI in bladder cancer involves lapatinib, afatinib and neratinib, which have shown promising results. In a phase II trial, lapatinib was used as second-line treatment for locally advanced or metastatic cell carcinoma with EGFR and/or HER2 overexpression (median time to progression (TTP) 8.6 weeks, mOS 17.9 weeks) [142]. The phase II trial of afatinib for platinum-refractory metastatic uroepithelial carcinoma demonstrated a significantly longer PFS in patients harboring HER2 or HER3 mutations than those without alterations (6.6 months vs 1.4 months, *P*<0.001) [143]. Dacomitinib, a second-generation of TKIs, not only inhibits HER2, HER4 and other associated proteins but also has the potential in mitigate resistance issues arising from bypass activation pathways such as HER2 [144]. An early study revealed effective inhibition of daclatinib in HER2-expressing bladder cancer cells [145], and a late-stage study is being prepared. A study investigating the efficacy of neratinib in patients with metastatic bladder cancer harboring a HER2-GRB7 gene fusion is underway.

##### ADCs

The currently approved ADC for uroepithelial carcinoma is RC48. T-DM1 and TDX-d are being studied. The results from a phase II trial, RC48-C005, demonstrated that RC48-ADC significantly improved objective remission rates and survival in patients with locally advanced or metastatic uroepithelial cancer(ORR 51.2%, mPFS 6.9 months, mOS 13.9 months) [41]. Based on this study, the FDA granted RC48 "Breakthrough Therapy Designation". Preliminary results from the latest study(RC48-C104) demonstrated an ORR of 80% for RC48 in combination with tremelimumab in previously untreated patients with first-line metastatic uroepithelial cancer. The ORR was also reported as follows based on the result of HER2 immunohistochemistry grouping: 100% for HER2-3+, 77.8% for HER2-2+, 66.7% for HER2-1+, and 50% for HER2-negative cases [146] . Therefore, RC48 was approved by CFDA for use in uroepithelial cancer treatment and was recommended by the CSCO guidelines. In preclinical research, T-DM1 was shown to demonstrate strong inhibitory effects on the growth of HER2-positive bladder cancer cell line RT4V6 [147]. A phase IB study, T-Dxd-A-U105, presented preliminary results at the 2022 American Society of Clinical Oncology ASCO congress. The combination of T-Dxd and nivolumab demonstrated significant efficacy in second- and later-line treatment of HER2-expressing urothelial carcinoma(DCR 76.6%, mPFS 6.9 months, mOS 11.0 months); however, it was associated with a notable incidence of serious adverse events (AEs ) [148]. While the combination of ADC and immune checkpoint inhibitors is a promising antitumor strategy, it is important to note that the side effects may be more severe.

#### Biliary tract cancer (BTC)

##### Antibodies

While no HER2-targeted drug has been approved for biliary malignancies yet, clinical trials have demonstrated the effectiveness of HER2-targeted therapy in gallbladder cancer. A phase II clinical trial found that trastuzumab had an ORR of 66.6% in the first or second-line treatment of patients with HER2-amplified gallbladder cancer [149]. Another phase IIA trial, MyPathway, demonstrated that combining trastuzumab with pertuzumab improved the treatment efficacy in patients with HER2-positive advanced metastatic biliary tract cancer(ORR 23%) [150]. As a result, the National Comprehensive Cancer Network (NCCN) guidelines recommend trastuzumab and pertuzumab for the treatment of advanced biliary tract tumors with HER2 amplification [151].The bispecific antibody ZW25 has been granted breakthrough therapy status by the FDA for patients with biliary tract cancer who have previously received other treatments and exhibit HER2 gene amplification. The findings from a phase I trial of Zanidatamab as a second-line therapy for biliary tract cancer were presented at the 2021 ASCO meeting, revealing an ORR of 40% and a DCR of 65% [152]. These results were significantly outperformed historical data on second-line chemotherapy. Currently, a global phase IIB study is underway [153].

##### TKIs

The reversible TKI, lapatinib, effectively inhibits the activation of MAPK, PI3K-AKT (protein kinase B), and phospholipaseCγ (PLCγ) downstream signaling pathways by blocking both HER1 and HER2 [154]. However, despite being evaluated in various clinical settings, lapatinib has not shown efficacy for the management of mUC [155–157]. Additionally, the TKI drug neratinib was shown to be safe and well-tolerable for the second-line treatment of HER2-mutated BTC patients(ORR 16%, mPFS 2.8 months, mOS 5.4 months) [158]. However, further research is needed to validate these findings.

##### ADCs

The exploration of ADC drugs in HER2-positive biliary tract cancer has shown promising results in recent years. A phase II study(HERB) was conducted by ASCO in 2022 to treat HER2-positive BTC patients (ORR 36.4%, DCR 81.8%, mPFS 5.7 months) [159]. Notably, positive outcomes were also observed in patients with low HER2 expression (ORR 12.5%, DCR 75%, mPFS 3.5 months) [159]. A combined analysis of two phase II clinical trials, namely RC48-C005 and RC48-C009, was conducted in a cohort of 107 patients with HER2-positive metastatic urothelial carcinoma (mUC) who had previously failed at least one line of systemic chemotherapy(ORR 50.5%, PFS 5.9 months) [160]. Trastuzumab duocarmazine (SYD985) is composed of trastuzumab, a cleavable linker, and duocarmycin (a DNA alkylator) [161]. Recently, a phase I trial assessed the safety and activity of SYD985 in advanced tumors patients with local or advanced urothelial cancer (partial response rate 25%) [67].

#### Colorectal cancer

##### Antibodies and TKIs

Research has shown that the use of HER2 monoclonal antibodies alone is not effective in treating colorectal cancer. However, combining monoclonal antibodies with TKIs has been found to be an effective approach. The combination of trastuzumab and pertuzumab was tested in a phase IIA trial (MyPathway) as first-line treatment for metastatic colorectal cancer (mCRC), Which resulting in an ORR of 32%. Consequently, the NCCN guidelines now recommend using trastuzumab combined with pertuzumab and lapatinib as initial therapies for mCRC [162]. Another phase II trial(HERACLES) demonstrated that lapatinib combined with trastuzumab was active and well tolerated in patients with refractory HER2-positive metastatic colorectal cancer (ORR 35%) [163]. The combination of tucatinib and trastuzumab demonstrated good efficacy in a phase II clinical trial (MOUNTAINEER) (ORR 55%, mPFS 6.2 months, OS 17.3 months) [164]. Similarly, the ongoing phase II trial (HER2-FUSCC-G) demonstrated that the combination of pyrotinib and trastuzumab also demonstrated promising antitumor activity(mPFS 7.53 months, OS 16.8 month) [165].

##### ADCs

The ADCs that have shown better efficacy in colorectal cancer patients include T-DM1 and T-dxd. According to a phase II clinical study called HERACLES-B, the combination of T-DM1 and pertuzumab has been shown to have low toxicity and high disease control rate (80% in 4 months) in the treatment of HER2-positive mCRC [165]. This combination is presents a viable option for patients with low tumor burden as an active anti-HER2 therapy, while T-DXd monotherapy has shown some antitumor activity in HER2-positive mCRC patients according to the phase II clinical trial (Destiny-CRC01) [166]. Therefore, NCCN guidelines recommend T-Dxd monotherapy as a treatment for mCRC patients with HER2 amplification. However, it is crucial to monitor and intervene in interstitial lung disease (ILD), which remains a potential adverse reaction among patients received T-DXd monotherapy.

#### Other malignancies

HER2 gene abnormalities (mutation/deletion/amplification) have alse been detected in ovarian cancer, cervical cancer, endometrial cancer, bladder cancer, and salivary gland cancer [167, 168]. However, the efficacy of classic HER2 monoclonal antibodies such as trastuzumab and pertuzumab is not satisfactory for treating uterine or ovarian carcinosarcoma. Preclinical trial data has shown that T-DM1, exhibits potent antitumor activity against chemotherapy-resistant epithelial ovarian cancer (EOC) with HER2-overexpressing [169]. In a phase II clinical trial NCI-MATCH, T-DM1 has demonstrated activity in patients with salivary gland tumors [168]. Additionally, the novel ADC drug SYD985 has shown promising results in preclinical research on uterine and ovarian carcinoma [170]. The specific efficacy of these treatments needs to be confirmed in subsequent clinical trials. The phase II trial DESTINY-PanTumor02 demonstrated that T-DXd exhibited favorable efficacy and safety profiles in various tumor types with HER2 overexpression, including patients with cervical cancer, endometrial cancer, ovarian cancer, biliary tract cancer, and bladder cancer (ORR 37.1%, DCR 83.2%, median duration of response 11.8 months). Importantly, patients with IHC3+ expression had a higher ORR of 61.3% and a longer DOR of 22.1 months [171]. Currently, there are several ongoing clinical trials related to this matter (Table 2).

## Conclusions and perspectives

HER2-targeted drugs have been effective at treating tumors for nearly two decades and have become a significant milestone in the field. Trastuzumab, a monoclonal antibody, represents the most advanced drug targeting anti-HER2 therapy and has gained approval the treatment of breast cancer(BC), GC and mCRC treatment [172]. The TKIs targeting HER2 are primarily utilized in BC, NSCLC, and mCRC. In contrast, ADCs have gained FDA approval for the treatment of breast cancer, bladder cancer, and NSCLC. They have also exhibited remarkable efficacy in the treatment of ovarian, bladder, gastric, colon, cervical, endometrial cancers as well as biliary tract cancer; thus holding a promising future.

However the presence of primary and acquired resistance presents significant clinical challenges. The mechanisms underlying primary resistance to anti-HER2 therapy include the following: (1) target receptor inactivation [173]; (2) abnormal activation of downstream components within the PI3K/Akt/mTOR and Ras-Raf-MAPK signaling pathways [174]; (3)overexpression of other HER ligands or receptors [175, 176]; (4) alternative signaling generated by other receptors, such as the insulin-like growth factor-1 receptor (IGF1R) [177]; and (5) the exertion of influence by the tumor microenvironment [178]. Recent studies suggest that miRNA-mediated alterations in gene expression are involve in the acquisition of drug resistance [179]. The membrane-associated glycoprotein mucin-4 (MUC4) may potentially mask the extracellular domain of HER2, thereby impeding effective binding with antibodies [180]. Acquired resistance primarily arises from alterations in the level of target signaling or active target receptors [181]. Furthermore, HER2 gene mutations can result in altered or enhanced interactions between HER2 and trastuzumab, which could manifest as either resistance or sensitivity [182, 183].

In response to resistance to HER2-targeted therapy, two main strategies have been employed. One approach involves the development of drugs with a novel structure and enhanced efficacy. For instance, lapatinib and pyrotinib can act on the intracellular domain of both the HER1 and HER2 proteins. And ADCs can cleave, internalize, and release payloads to HER2-positive cancer cells through their special structure, resulting in improved efficacy. Another strategy focuses on exploring drug combinations to overcome drug resistance, such as utilizing extracellular domain-binding trastuzumab and TKIs in breast and colon cancer treatment. Additionally, the combination of inhibitors targeting the PI3K-AKT-mTOR pathway or immune checkpoints with anti-HER2 agents has demonstrated promising results [184, 185]. A recent study showed that inhibiting the EGFR/HER2 signaling network affects cancer-associated fibroblasts (CAFs) in pancreatic ductal adenocarcinoma (PDAC) organoids and mouse models. Specifically, it reveals that activated myofibroblastic CAFs (myCAFs) through EGFR play a crucial role in promoting PDAC metastasis in mice. These findings highlight the importance of anti-HER2 therapy and immunotherapy as potential treatment strategies for PDAC [186]. A similar strategy can be applied for acquired resistance. Moreover, the HER2 status should be retested to predict the effect of HER2-targeted therapy.

The determination of HER2 status plays a crucial role in the appropriateness of HER2-targeted therapy. However, there is currently a lack of consensus and standardized definitions for HER2-positive across different tumors, thus breast cancer serves as the reference criterion. The diagnostic criteria included immunohistochemical(IHC)+++ or IHC++ and fluorescence in situ hybridization (FISH)-positive. It is important to note that HER2 overexpression and amplification patterns vary among different tumors [67, 187], resulting in varying levels of effectiveness of anti-HER2 therapy in patients with different cancer types. Therefore, there is an urgent need to establish detailed positive criteria for different tumors. Furthermore, performing repeated biopsies to assess HER2 status during disease progression is crucial. The inaccessibility of lesion biopsy remains a challenge in current clinical progress. Clinical trials led by the DESTINY series have recently used liquid biopsy techniques such as ctDNA to detect the HER2 status of patients [188]. This noninvasive method captures the precise expression profile of dynamic and heterogeneous tumor genomes while mitigating the risks associated with bleeding, infection, and tumor dissemination caused by traditional needle biopsy [188]. However, issues such as detection accuracy, positive cutoff value, and cost need to be resolved.

HER2-targeted therapy is a valuable antitumor treatment that merits further enhancement and advancement. While several mechanisms of resistance have been identified, the impact of new drug combinations on resistance profiles has not been determined [189–193]. However, further research is needed to explore the underlying mechanism of these drugs resistance and develop more effective drugs and treatment regimens.

## Data Availability

The datasets generated during the current study are available in the cBioPortal repository, cBioPortal for Cancer Genomics.

## Abbreviations

ADCs: Antibody-drug conjugates
ADCC: Antibody-dependent cell-mediated cytotoxic effects
AKT: Protein kinase B
BC: Breast cancer
CSCO: Chinese society of clinical oncology
CAFs: Cancer-associated fibroblasts
EGFR: Epidermal growth factor receptor
ECD: Extracellular structural domain
EOC: Epithelial ovarian cancer
ERK: Extracellular signal-regulated kinase
FDA: Food and Drug Administration
FISH: Fluorescence in situ hybridization
GC: Gastric cancer
G/GEJ: Gastric/gastroesophageal junction
GEA: Gastroesophageal cancer
GTP: Guanosine triphosphate
GDP: Guanosine diphosphate
GRB2: Growth factor receptor-bound protein 2
HER2: Human epidermal growth factor receptor2
IHC: Immunohistochemical
ILD: Interstitial pneumonia
mTORC: Mammalian target of rapamycin
mAbs: Monoclonal antibodies
mCRC: Metastatic colorectal cancer
MEK: Mitogen-activated extracellular signal-regulated kinase
MUC4: Membrane-associated glycoprotein mucin-4
NSCLC: Non-small cell lung cancer
NCCN: National comprehensive cancer network
OS: Overall survival
ORR: Objective remission rate
PFS: Progression-free survival
PTEN: Phosphatase and tensin homolog
PDK1: Phosphoinositide-dependent protein kinase 1
PDAC: Pancreatic ductal adenocarcinoma
PLC: Phospholipase C
PKC: Protein kinase C
SOS: Guanosine release protein
STAT: Signal transducer and activator of transcription
TKI: Tyrosine kinase inhibitors
TTP: Time to progression
TMD: Transmembrane domain
TSC1/2: Tuberous sclerosis complex1/2
UC: Urothelial carcinoma
